# Non-Destructive Assessment of Gamma Radiation Aging in Nuclear Cables via New Dielectric Spectroscopy Markers and Machine Learning Algorithm

**DOI:** 10.3390/polym18040500

**Published:** 2026-02-17

**Authors:** Ahmad Abualasal, Zoltán Ádám Tamus

**Affiliations:** Department of Electric Power Engineering, Faculty of Electrical Engineering and Informatics, Budapest University of Technology and Economics, H-1111 Budapest, Hungary; aahmadabualasal@edu.bme.hu

**Keywords:** dielectric spectroscopy, nuclear cables, gamma irradiation, condition monitoring, non-destructive testing, ethylene propylene rubber, chlorosulfonated polyethylene

## Abstract

Low-voltage instrumentation and control (I&C) cables in nuclear power plants are continuously exposed to gamma (γ) radiation within containment areas, leading to cumulative degradation of their polymer insulation over decades of operation. Since conventional mechanical aging assessments are destructive, this study establishes a non-destructive diagnostic framework using high-frequency dielectric spectroscopy. Cable samples with ethylene propylene rubber (EPR) insulation and chlorosulfonated polyethylene (CSPE) jackets were subjected to controlled γ-irradiation at doses up to 1200 kGy. The broadband dielectric response was analyzed along with derived novel diagnostic parameters from capacitance and loss tangent spectra and a machine learning AI algorithm. The results show a strong, material-dependent relationship between radiation dose and dielectric indicators. For EPR insulation, the central capacitance (CC) and (C × F × LF) exhibit high positive sensitivity for Black and White EPR materials, respectively, whereas for CSPE jackets, the central frequency (CF) shows a pronounced monotonic decrease with the radiation exposure. These findings enable a straightforward, transparent interpretation of dielectric data and implement a new, accurate method of irradiated cables diagnosis.

## 1. Introduction

Instrumentation and control (I&C) low-voltage cables are critical for the safe operation of nuclear power plants (NPPs), functioning as their operational veins. With many NPPs now pursuing life extensions from 30–40 years up to 60–80 years [1], managing cable aging has become a major concern. The International Atomic Energy Agency (IAEA) highlights cable aging as a key issue and provides guidance through its International Generic Ageing Lessons Learned (IGALL) program [2], while benchmark analyses compare available condition monitoring techniques [3]. The cable polymer insulation deteriorates due to the cumulative effect of various stress factors, such as thermal, radiation, mechanical, and electrical stresses, which induce chemical reactions like oxidation, chain scission, and cross-linking reactions [4,5]. That is, the molecular-level changes mentioned above take place directly in the electrical properties of the insulation. More specifically, the oxidation reaction leads to the formation of polar carbonyl groups, resulting in increased permittivity and higher dielectric losses. Chain scission slightly increases these values, whereas cross-linking tends to decrease them by reducing molecular motion. Engineers measure the changes in capacitance and tan delta values as non-destructive indicators of insulation aging.

Radiation exposure, particularly γ-rays in oxygen, aggressively attacks polymer structures, accelerating oxidative degradation and embrittlement [6,7]. Comparative studies show that both gamma and beta irradiation cause similar degradation trends, increasing dielectric permittivity while reducing tensile elongation [8]. Thermal aging also deteriorates polymers through thermal-oxidative reactions, altering dielectric properties, as seen in crosslinked polyethylene (XLPE) and nuclear-grade cable materials [9,10,11]. A common industry end-of-life criterion is a 50% reduction in elongation at break (EaB), a key mechanical indicator of aging [3]. However, EaB measurement is destructive, driving the need for non-destructive condition monitoring techniques.

Various non-destructive electrical methods have been investigated [3,12], including line resonance analysis (LIRA), which is very susceptible and not directly designed to investigate the insulation material health [13], and reflectometry techniques that detect discrete faults and damages rather than the uniform aging of the insulation polymer itself [14]. Semi-invasive methods like the indenter modulus test (IEC 62582-2) also exist but cause minor damage [3]. Each technique has limitations, especially for cables undergoing combined degradation in nuclear environments [4,12]. Measuring complex permittivity over a frequency range has emerged as a particularly promising tool despite its complexity [15]. Verifying the adequacy of the dielectric spectroscopy with destructive techniques; studies on XLPE cables show that aged samples exhibit increased real and imaginary permittivity, correlating strongly with the decline of destructive EaB and oxidation levels [16]. Similar results are reported for EPR-based nuclear cables under thermal and gamma aging, where low-frequency permittivity increases systematically with aging dose, mirroring EaB reduction [17,18]. For PVC insulation, the “central frequency” and “central loss factor” of the dielectric loss peak correlate strongly (R > 0.85) with thermal aging [19]. A recent study on EPR/CSPE cables (the polymer used in this research) evaluated eight dielectric-derived parameters, many showing strong, monotonic trends with radiation dose that paralleled EaB drop [20].

Multi-technique approaches validate dielectric diagnostics. Studies combining dielectric spectroscopy with physiochemical analyses (FTIR, DSC, micro-indentation) confirm dielectric shifts that correlate with carbonyl buildup and increased stiffness [21,22,23]. Modeling efforts further agree with this approach, using probabilistic degradation models and frameworks for combined radiation-thermal stress to link dielectric parameters to aging mechanisms [24,25]. Field implementation is advancing, with portable dielectric spectroscopy devices operating in the 1–200 kHz range for live on-site measurements [26]. Very low-frequency (VLF) tan δ methods have also proven effective for on-site cable life assessment even outside nuclear applications, as in photovoltaic cables [27,28].

Despite progress, reliably correlating electrical diagnostics with traditional aging criteria for nuclear safety remains challenging. Most research focuses on power cables (XLPE, EPR) under thermal or combined stresses, with fewer studies on I&C cables with CSPE/EPR construction under pure radiation aging [29,30]. I&C cables experience lower temperatures but prolonged radiation, making their aging behavior distinct. Our group’s recent work on low-voltage EPR/CSPE cables showed that dielectric spectroscopy, extended voltage response (EVR), and polarization–depolarization current measurements are highly sensitive to both irradiation and thermal aging; meanwhile, capacitance and tan δ correlated strongly with residual EaB [29,30,31].

Thermal aging studies show analogous trends, with tan δ and EVR responses shifting significantly and aligning well with EaB reduction for both EPR and CSPE [30]. Furthermore, aging mode affects diagnostic responses; for instance, low-frequency dielectric properties of CSPE/XLPE cables degrade faster under combined heat and mechanical bending than heat alone [32]. Other studies apply non-destructive techniques to irradiated low-voltage cables and compare properties after thermal and thermo-mechanical aging, while reviews detail radiation-induced effects on insulation performance [33,34,35].

In this paper, a new reliable insulation diagnosis method is proposed for irradiated I&C cables based on non-destructive dielectric measurements. The study targets a typical LV instrumentation cable with EPR insulation and CSPE jacket, subjected to controlled γ-irradiation aging. The sample is a two-core low-voltage I&C cable with ethylene propylene rubber insulation and a chlorosulfonated polyethylene jacket; a configuration widely used in nuclear installations due to its fire resistance and compliance with IEEE 383 standards [2,3]. The cable was selected because both EPR and CSPE exhibit distinct but well-documented degradation pathways under radiation [7], allowing comparative analysis of insulation vs. jacket aging on one hand and, on the other hand—most importantly—addressing a new method for aging diagnosis. By analyzing the frequency-domain dielectric response of the aged cable segments, the diagnostic formulas that exhibit the most pronounced and consistent changes can be identified using the Gaussian process regression (GPR)—AI machine learning model—to evaluate their efficacy as proper aging indicators. This work builds upon prior research while addressing the gap for radiation-aged I&C cables, aiming to validate a new, practical, sufficiently accurate and non-destructive approach to infer the radiation aging state from dielectric measurements.

## 2. Materials and Methods

The measured dielectric spectroscopy data have already been published [31]; here are the most critical details affecting the results, i.e., the samples, the aging parameters, and the measurement method are presented.

### 2.1. Cable Sample

The analyzed specimen was a low-voltage (LV) instrumentation and control (I&C) cable, commonly used in nuclear power plants (NPPs). The two-core cable featured ethylene propylene rubber (EPR) inner insulation with a thickness of 0.76 mm and a conductor radius of 0.732 mm. Its outer sheath was composed of chlorosulfonated polyethylene (CSPE), with a total external diameter of 12.5 mm. The cable, manufactured by JS Cable Co., Ltd. (Seoul, Republic of Korea), was designed for Class 1E applications with a maximum rated operating temperature of 90 °C. For this study, five cable samples were prepared, each measuring 0.5 m in length.

### 2.2. Aging Protocol

Accelerated aging was performed using γ-ray irradiation (^60^Co source) at the Institute of Isotopes Co. Ltd. (Budapest, Hungary). The aging process was performed with a dose rate of 0.8 kGy/h at 25 °C with continuous air ventilation to prevent ozone buildup. Five cable samples were irradiated with absorbed doses of 0 (unaged), 120, 360, 600, 840, and 1200 kGy to simulate the long-term operational aging process. These doses correspond to cumulative service exposure durations of 14, 48, 113, and 162 years, assuming a typical background dose rate of 1 Gy/h in containment.

### 2.3. Dielectric Measurement

**Figure 1:**
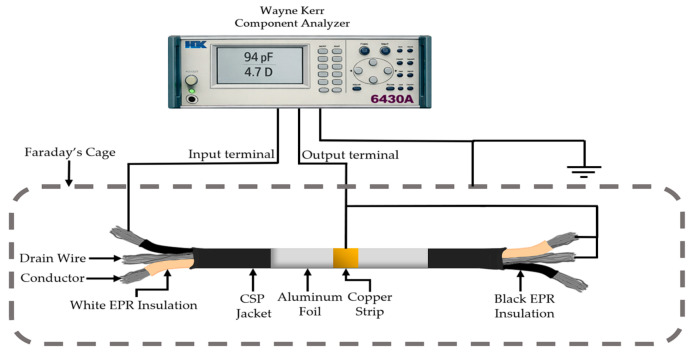
Measurement configuration.

**Figure 2:**
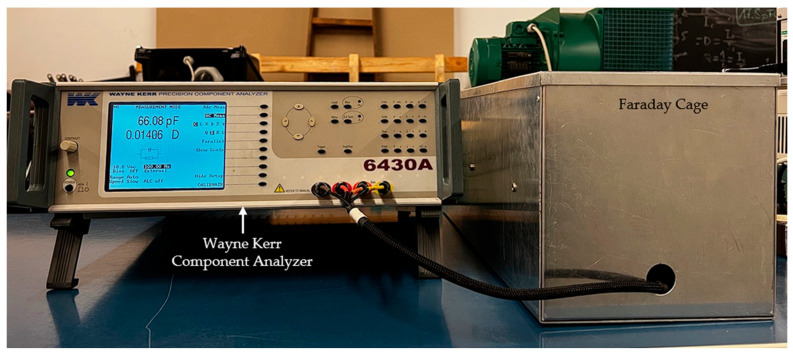
Measurement setup in the laboratory.

Broadband dielectric measurements were carried out in the frequency domain using a Wayne Kerr 6430A device, Wayne Kerr Electronics, Bognor Regis, UK. Measurements focused on the 2 kHz–500 kHz high-frequency band. An input signal of 5 V_rms_ was applied to the cable conductor, while the output was obtained from the drain wire. For insulation measurements, the jacket and opposing conductor were shorted. For the jacket, an aluminum foil electrode (29 cm-long) was wrapped around the cable’s outer surface, receiving the input signal, with the output again extracted via the drain (Figure 1). All measurements were performed inside a Faraday cage to eliminate external electromagnetic interference (Figure 2).

### 2.4. Calculation of Derived Quantities

The dielectric properties—capacitance and dissipation factor (tan δ)—were measured at multiple frequencies. From these, similarly to [19], a series of derived dielectric quantities was computed, including the following:

#### 2.4.1. Central Loss Factor (CLF)

Equation (1) illustrates how the CLF was determined by adding up the logarithm of the frequency times the measured tan δ values at certain frequencies and then dividing this total by the logarithm of the frequencies.

(1) $$CLF=\frac{\sum_{i=1}^{n}\log f_{i}\times tan\delta_{i}}{\sum_{i=1}^{n}\log f_{i}}$$

#### 2.4.2. Central Frequency (CF)

Equation (2) illustrates how the CF was determined by adding up the logarithm of the frequency times the measured tan δ values, dividing this total by the total of the measured tan δ values, and using this as an exponent to base 10.

(2) $$CF=10^{\left(\frac{\sum_{i=1}^{n}\log f_{i}\times tan\delta_{i}}{\sum_{i=1}^{n}tan\delta_{i}}\right)}$$

#### 2.4.3. Central Capacitance (CC)

Central capacitance (CC) was obtained by the same procedure as the CLF set of values, just substituting tan δ with the capacitance values, as Equation (3) shows.

(3) $$CC=\frac{\sum_{i=1}^{n}\log f_{i}\times C_{i}}{\sum_{i=1}^{n}\log f_{i}}$$

#### 2.4.4. Capacitance × log (Frequency) × tan δ (C × logF × LF)

Another derived quantity, the product of capacitance, the logarithm of frequency, and tan δ (C × lgF × LF), was calculated as shown in Equation (4). This was done by multiplying the logarithm of the frequencies by the corresponding loss factor and capacitance values for each frequency and then summing these products.

(4) $$C\times logF\times LF=\overset{n}{\underset{i=1}{\sum }}\log f_{i}\times tan\delta_{i}\times C_{i}$$

#### 2.4.5. Capacitance × Frequency × tan δ (C × F × LF)

The (capacitance × frequency × tan δ) (C × F × LF) was derived similarly to Equation (4); however, instead of the logarithms of the frequencies, the actual measured frequencies were used, as Equation (5) shows.

(5) $$C\times F\times LF=\overset{n}{\underset{i=1}{\sum }}f_{i}\times tan\delta_{i}\times C_{i}$$

#### 2.4.6. Area of Capacitance Times tan δ at Logarithmic Frequencies (Alog)

A similarly-derived formula to the previous one was calculated in this case by essentially integrating the tan δ multiplied by the values of the capacitance over the logarithmic frequency range. This integration was implemented by calculating the area of rectangles and summing these areas, as shown in Equation (6).

(6) $$Alog=\sum_{i=1}^{n}\left[\min\left(p_{i},p_{i+1}\right)+\max\left(p_{i},p_{i+1}\right)-\frac{\min\left(p_{i},p_{i+1}\right)}{2}\right]\times \left[\left.\log(\frac{f_{i+1}}{f_{i}})\right]\right.\;$$

#### 2.4.7. Multiplication of Available Values: CF × CLF × CC, CF × CLF

By multiplying the CF, CC, and CLF values, two additional derived quantities (CF × -CLF × CC and CLF × CC) were computed.

### 2.5. Data Analysis Approach

In this study, the analysis focused on assessing the relationship between the derived dielectric quantities and the aging dose (in kGy), rather than mechanical degradation indicators like elongation at break (EaB), as done in prior work. The GPR model was deployed for each dielectric parameter against the corresponding absorbed dose, with subsequent data behavior comparisons to determine the strength and predictability of each parameter. This approach allowed for identifying which electrical indicators most reliably track radiation-induced degradation trends across the EPR and CSPE insulation materials.

## 3. Results

The results are illustrated for each of the cable insulation components, as shown in Table 1. Eight of the derived quantities are listed in the first row for the unaged sample. Successively, the same quantities are also presented in the following rows for each sample, with an additional dose amount step by step until the readings for the 1200 kGy were achieved. For both the inner and outer insulation, the values of CF generally decrease with the increase in the radiation dose, unlike the other values that significantly rise with absorbed dose. However, when it comes to the jacket insulation, two quantities break the trend and go inversely with the dose: CLF × CF × CC and CF × C LF.

## 4. Analysis of Results

To address the nonlinear degradation kinetics of polymer materials under gamma irradiation, GPR was employed as a Bayesian non-parametric framework for modeling the dose-response relationships. GPR represents a probabilistic approach that places a prior distribution over possible functions and updates this to a posterior distribution conditioned on the observed dielectric parameter data, thereby providing not only mean predictions but also full uncertainty quantification through predictive variances [36]. Sensitivity to material-specific degradation behavior is preserved while overusing other parameters is mitigated by this approach, which is well-suited to small datasets such as the six dose points per material examined here [37]. Both the monotonic degradation observed in ethylene propylene rubber EPR insulation and the non-monotonic response characteristic of chlorosulfonated polyethylene CSPE jackets are captured by this method. Within the GPR framework, a radial basis function was selected for EPR and CSPE, with hyperparameters optimized via maximum marginal likelihood to reflect the distinct degradation mechanisms associated with each polymer chemistry [38]. Degradation rates with associated confidence intervals are enabled by applying GPR to dielectric spectroscopy data through modeling smooth functional relationships between absorbed dose and derived dielectric parameters without imposing a predefined parametric form. Full predictive distributions with quantified uncertainty are generated by GPR, addressing the key limitations of conventional correlation methods while interpretability is maintained through kernel parameters directly linked to degradation physics [39,40].

### 4.1. Mathematical Model

Based on the literature that profoundly discussed the GRP mathematical model, it was possible to apply to this study as a highly predictive, reliable alternative [37,38,39,40]. The experimental data were added to the GPR algorithm using artificial intelligence to make a mathematical model that calculates the required aging parameters. The procedure involved the following steps:

1. Defining the training dataset $\left(X,y\right)$:

For each dielectric parameter, the general dataset can be expressed as in Equation (7):

(7) $$D={\left\{\left(x_{i},y_{i}\right)\right\}}_{i=1}^{N},\;N=6\;$$

where:

$x_{i}$ = absorbed dose [kGy].

$y_{i}$ = the measured value of the chosen dielectric parameter at dose $x_{i}$.

In this study, the input vector is fixed and identical for all parameters as shown in Equation (8):

(8) $$X=\left[\begin{array}{c} x_{1}\\ x_{2}\\ :\\ :\\ x_{6} \end{array}\right]=\left[\begin{array}{c} 0\\ 120\\ :\\ :\\ 1200 \end{array}\right]\;[\mathrm{kGy}]\;$$

Correspondingly, the dielectric parameter (CF, CC, ALog, …) has its own output vector portrayed in Equation (9):

(9) $$y=\left[\begin{array}{c} \begin{array}{c} y_{1}\\ y_{2} \end{array}\\ :\\ :\\ y_{6} \end{array}\right]\;$$

where $\left(y_{1},\ldots ,y_{6}\right)$ are the experimentally and then mathematically computed parameter values at each of the dose values.

At this point, all the GPR algorithm inputs from the absorbed dose were inserted, and the corresponding parameter values were observed.

2. Stating the measurement model:

Each observed dielectric value is assumed to be a noisy observation of an underlying smooth degradation function $f\left(x\right)$:

(10) $$y_{i}=f\left(x_{i}\right)+\varepsilon_{i},\varepsilon_{i}∼N\left(0,\sigma_{n}^{2}\right)\;$$

$f\left(x\right)$: A true but unknown dose-response curve for this parameter.

$\varepsilon_{i}$ = measurement + processing noise.

$\sigma_{n}^{2}$ = noise variance (estimated from the data).

3. Placing a Gaussian process prior over the unknown function (Equation (11)):

Instead of assuming a specific parametric form (linear, exponential, etc.), GPR assumes:

(11) $$f\left(x\right)∼GP\;(m\left(x\right),\;k\left(x,x^{\prime }\right))\;$$

meaning that the function values at any set of inputs are jointly Gaussian.

$m\left(x\right)$: mean function (often set to 0 after centering/normalization).

$k\left(x,x^{\prime }\right)$: covariance (kernel) that controls smoothness and correlation.

Before seeing the data, GPR assumes that many possible smooth dose-response curves are plausible; the kernel describes how “smooth” or “flexible” those curves are allowed to be.

4. Building the kernel covariance matrix K (Equation (12)) using the dose values:

Using the training dose values, the kernel matrix $K\in {\mathbb{R}}^{6\times 6}$ is constructed elementwise:

(12) $$K_{ij}=k\left(x_{i},x_{j}\;|\;l,\sigma_{f}^{2}\right)\;$$

l (length scale): controls how quickly the function can vary with dose; small l implies rapid dose sensitivity, while larger l indicates a smoother and slowly varying aging trend.

$\sigma_{f}^{2}$ (signal variance): magnitude of systematic dose-dependence.

Written explicitly (Equation (13)):

(13) $$K=\left[\begin{array}{cccc} k\left(x_{1},x_{1}\right) & k\left(x_{1},x_{2}\right) & \ldots & k\left(x_{1},x_{6}\right)\\ k\left(x_{2},x_{1}\right) & k\left(x_{2},x_{2}\right) & \ldots & k\left(x_{2},x_{6}\right)\\ \vdots & \vdots & \ddots & \vdots \\ k\left(x_{6},x_{1}\right) & k\left(x_{6},x_{2}\right) & \ldots & k\left(x_{6},x_{6}\right) \end{array}\right]$$

If two doses are close in value (e.g., 600 and 840), most kernels assign a higher correlation than doses far apart (e.g., 0 and 1200). This enforces smoothness across doses.

5. Adding the noise term to obtain $K_{y}$:

Because measurements are noisy, the covariance (Equation (14)) of the observed vector y is:

(14) $$K_{y}=K+\sigma_{n}^{2}I$$

Explicitly shown in Equation (15):

(15) $$K_{y}=\left[\begin{array}{cccc} k\left(x_{1},x_{1}\right)+\sigma_{n}^{2} & k\left(x_{1},x_{2}\right) & \ldots & k\left(x_{1},x_{6}\right)\\ k\left(x_{2},x_{1}\right) & k\left(x_{2},x_{2}\right)+\sigma_{n}^{2} & \ldots & k\left(x_{2},x_{6}\right)\\ \vdots & \vdots & \ddots & \vdots \\ k\left(x_{6},x_{1}\right) & k\left(x_{6},x_{2}\right) & \ldots & k\left(x_{6},x_{6}\right)+\sigma_{n}^{2} \end{array}\right]$$

The diagonal inflation $\sigma_{n}^{2}$ tells the model that each measured dielectric value contains uncertainty; without it, GPR would attempt to interpolate the data exactly.

6. Identifying the hyperparameters and where they appear:

Equation (16) includes the hyperparameters that the kernel depends on as the set θ:

(16) $$\theta =\left\{l,\sigma_{f}^{2},\sigma_{n}^{2}\right\}\;$$

where:

$\sigma_{n}^{2}$ (noise variance): scatter not explained by a smooth dose response.

These hyperparameters are substituted:

l and $\sigma_{f}^{2}$ appear inside $k\left(x_{i},x_{j}\right)$, hence inside K

$\sigma_{n}^{2}$ appears in $K_{y}=K+\sigma_{n}^{2}I$

7. Training the model by maximizing the log marginal likelihood (Equation (17)), GPR determines θ by maximizing:

(17) $$\log p\left(y|X,\theta \right)=-\frac{1}{2}y^{T}K_{y}^{-1}y-\frac{1}{2}log|K_{y}|-\frac{N}{2}\log\left(2\pi \right)$$

where:

$-\frac{1}{2}y^{T}K_{y}^{-1}y$: rewards fitting the measured dielectric values well.

$-\frac{1}{2}log|K_{y}|$: to avoid overly complex functions that fluctuate just to satisfy the data points.

$-\frac{N}{2}\log\left(2\pi \right)$: constant term

8. Prediction of the dielectric parameter at a new dose $x_{*}$:

To estimate the parameter defined by Equation (18) at any new dose (e.g., $x_{*}=500$ [kGy]), compute the covariance vector between $x_{*}$ and the training doses:

(18) $$k_{*}=\left[\begin{array}{c} k\left(x_{*},x_{1}\right)\\ k\left(x_{*},x_{2}\right)\\ \vdots \\ k\left(x_{*},x_{6}\right) \end{array}\right]=\left[\begin{array}{c} k\left(x_{*},0\right)\\ k\left(x_{*},120\right)\\ k\left(x_{*},360\right)\\ k\left(x_{*},600\right)\\ k\left(x_{*},840\right)\\ k\left(x_{*},1200\right) \end{array}\right]$$

9. Finding predictive mean and predictive variance:

The posterior predictive mean (best estimate) can be calculated using Equation (19):

(19) $$\mu \left(x_{*}\right)=k_{*}^{T}K_{y}^{-1}y\;$$

And the posterior predictive variance (uncertainty) is defined by Equation (20):

(20) $$\sigma^{2}\left(x_{*}\right)=k\left(x_{*},x_{*}\right)-k_{*}^{T}K_{y}^{-1}k_{*}\;$$

where in the case study:

$\mu \left(x_{*}\right)$ is the predicted CF (or CC, etc.) at dose $x_{*}$

$\sigma \left(x_{*}\right)$ increases where data are sparse (often near the ends of the dose range)

10. Constructing the 95% confidence interval used in the figures:

Because the predictive distribution is Gaussian as in Equation (21):

(21) $$y\left(x_{*}\right)∼N\;\left(\mu \left(x_{*}\right),\sigma^{2}\left(x_{*}\right)\right)\;$$

the 95% confidence interval can be evaluated by Equation (22):

(22) $$\mu \left(x_{*}\right)\pm 1.96\;\sigma \left(x_{*}\right)\;$$

This interval is plotted as the shaded band around the GPR mean curve for each dielectric indicator in the figures that follow in the next pages.

11. Apply the same pipeline to every dielectric indicator and material:

The full procedure (Steps 1–10) is repeated for each derived dielectric parameter (CF, CC, CLF, ALog, etc.) and for each material subset (EPR black, EPR white, CSPE jacket). Therefore:

The input vector X remains the same as the absorbed dose values, while only the output vector y changes depending on the chosen dielectric parameter and material.

The previous GPR steps were applied to each of the dose values of the tested cables, and the results are illustrated in the following sections accordingly.

### 4.2. Black Core Insulation

**Figure 3:**
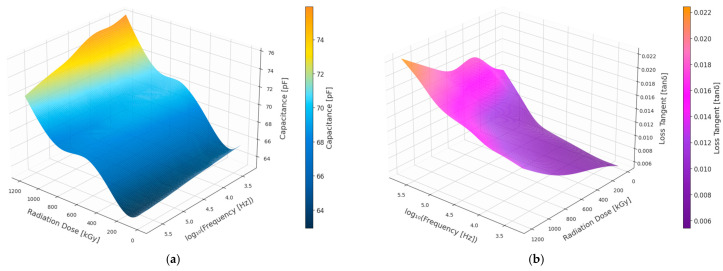
3D plot of Black EPR insulation. (**a**) Capacitance. (**b**) Dielectric loss (tan δ).

The three-dimensional plot in Figure 3 illustrates the effect of gamma radiation dose and frequency on the dielectric properties of the black EPR insulation material. The surface plot of capacitance indicates a monotonic increase with increased doses of radiation, and this is more dominant in the lower frequency region. The surface plot of the loss tangent (tan δ) also indicates a dose-dependent increase, which is more dominant in the lower frequency region. In contrast, both properties show attenuated sensitivity to dose at higher frequencies. Thus, the figure demonstrates that radiation aging fundamentally compromises the electrical fingerprint of degradation emerging in the low-frequency domain.

**Figure 4:**
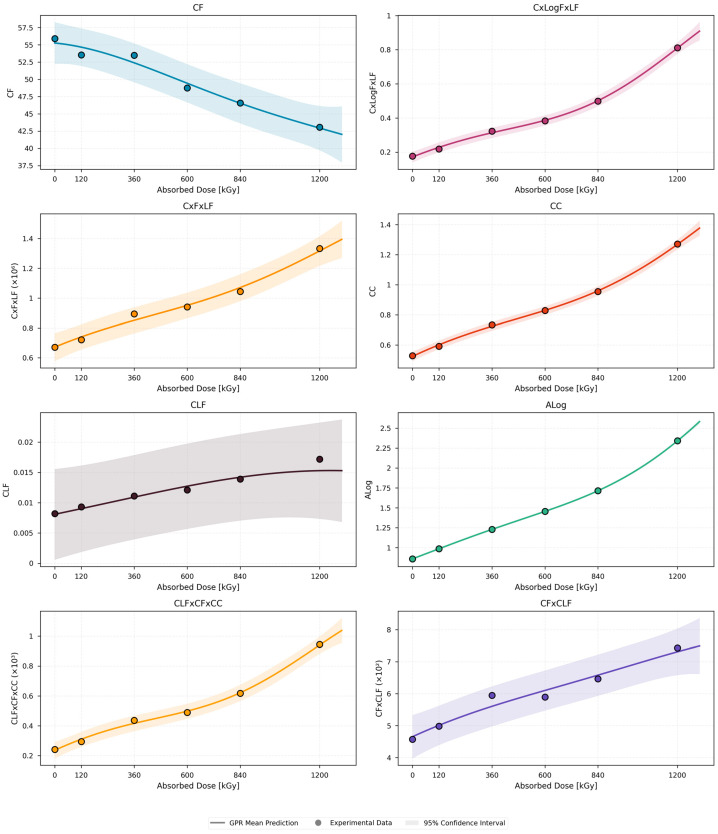
Black EPR dielectric quantities dose-dependent behavior using the GPR model.

Figure 4 presents the GPR mean predictions together with the experimental data points and the corresponding 95% confidence intervals for all evaluated dielectric indicators. Confidence interval indicates the uncertainty of the GPR predictions, with narrower intervals reflecting higher predictive confidence and model stability. In other words, there is a 95% probability that the true value of the predicted quantity lies within the shaded interval around the mean prediction.

Across all dielectric parameters, clear and physically consistent dose-dependent trends are observed. A systematic shift of dielectric loss toward lower frequencies as radiation aging progresses is reflected by the monotonic decrease in the central frequency CF with increasing absorbed dose. The cumulative growth of dielectric losses, enhanced polarization effects and increased permittivity dispersion driven by radiation-induced oxidation and microstructural degradation are reflected by the monotonic increases with dose exhibited by C × LogF × LF, C × F × LF, CC, CLF, ALog and CLF × CF × CC, enhanced polarization effects, and increased permittivity dispersion driven by radiation-induced oxidation and microstructural degradation. Mild nonlinear behavior in several parameters is captured by GPR models, particularly at higher dose levels ≥ 840 kGy, where departures from simple monotonic trends become apparent. An acceleration of degradation mechanisms at advanced stages of radiation exposure is suggested by these nonlinearities, which are most pronounced for C × F × LF, ALog, and CLF × CF × CC.

### 4.3. White Core Insulation

**Figure 5:**
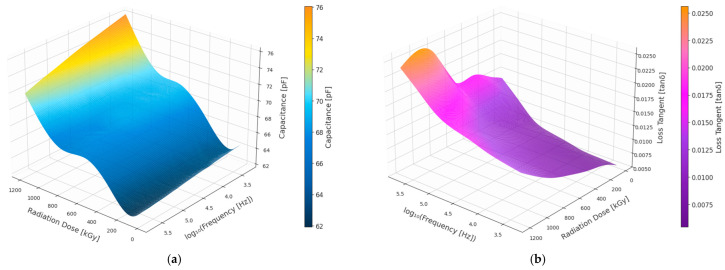
3D plot of White EPR insulation. (**a**) Capacitance. (**b**) Dielectric loss (tan δ).

Figure 5 presents a three-dimensional map of the response of the white EPR insulation, showing that both capacitance and loss tangent increase progressively with absorbed dose across the full frequency range, with the most pronounced effects occurring at lower frequencies. The radiation-induced changes dominate over variations arising from frequency tuning alone, even though both properties exhibit measurable frequency dependence. These combined dose and frequency-dependent trends closely mirror those previously observed in black EPR insulation.

**Figure 6:**
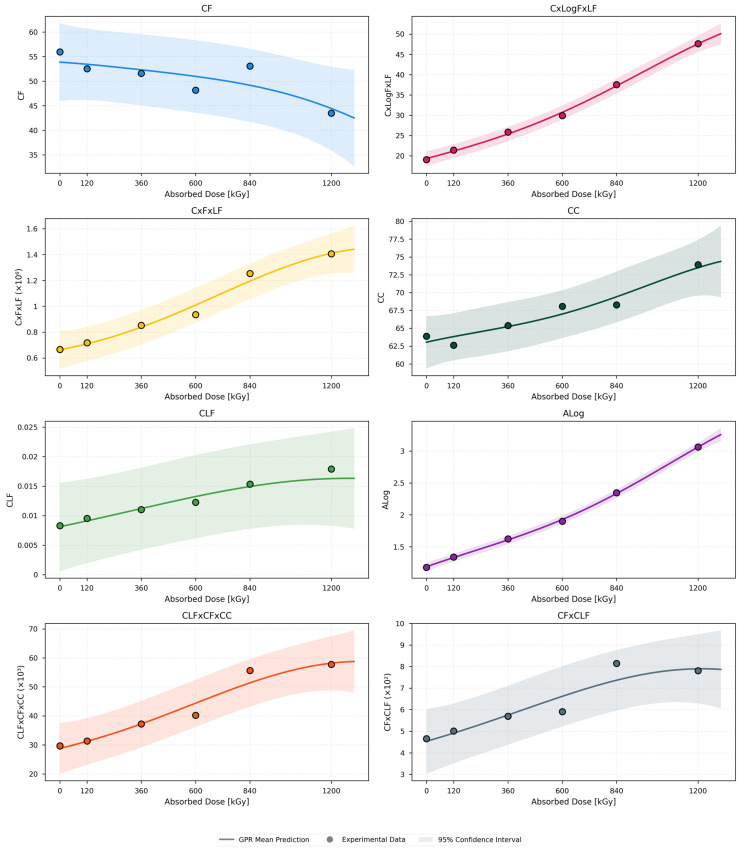
White EPR dielectric quantities dose-dependent behavior using the GPR model.

As illustrated in Figure 6, the experimental data are closely tracked by the Gaussian process regression (GPR) mean predictions, while narrow 95% confidence intervals are maintained, indicating strong model fidelity and well-constrained uncertainty. A systematic shift of dielectric loss toward lower frequencies as radiation aging progresses is reflected by the gradual monotonic decrease in the central frequency CF with increasing absorbed dose. Pronounced monotonic increases with dose are shown by all remaining parameters C × LogF × LF, C × F × LF, CC, CLF, ALog, CLF × CF × CC, and CF × CLF.

Particularly smooth nonlinear growth is displayed by C × LogF × LF ALog and C × F × LF among these parameters, and this behavior is effectively captured by the GPR models. A cumulative increase in dielectric losses and enhanced polarization activity with continued irradiation is suggested by this behavior. A stable and steadily increasing trend is followed by the central capacitance CC, consistent with radiation-induced permittivity enhancement, while CLF increases more gradually, indicating a progressive but less abrupt rise in overall dielectric dissipation.

Composite parameters such as CLF × CF × CC and CF × CLF further emphasize the coupled evolution of frequency shift and loss mechanisms. Their nonlinear behavior is successfully resolved by the GPR framework, particularly at higher dose levels, highlighting its ability to capture interacting degradation processes under advanced radiation exposure.

### 4.4. CSPE Jacket

**Figure 7:**
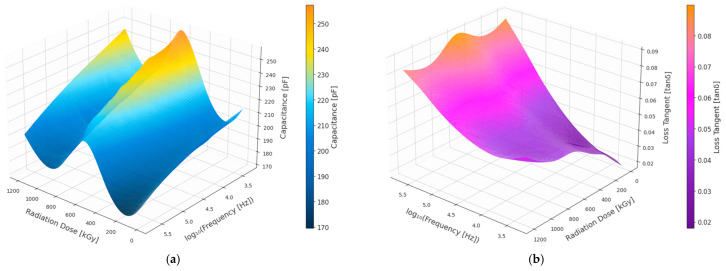
3D plot of Jacket CSPE insulation. (**a**) Capacitance. (**b**) Dielectric loss (tan δ).

According to the numerical results, distinct non-monotonic behavior is exhibited by both capacitance and loss tangent as the radiation dose increases. At 10 kHz, as illustrated in Figure 7, the capacitance increases from approximately 210 pF in the unaged state to 248 pF at 600 kGy, then decreases to 214.3 pF at 840 kGy before increasing again to 235 pF at the maximum dose of 1200 kGy. A similar trajectory is followed by the loss tangent, which increases from 0.0261 to 0.0430, then slightly decreases to 0.0424 before rising to 0.0512 at the highest dose.

This rise–dip–rise pattern is observed consistently across all measured frequencies, indicating that the most pronounced fluctuations occur at intermediate dose levels 360–840 kGy. A re-intensification of degradation mechanisms at advanced radiation exposure is suggested by the renewed increase in both parameters at 1200 kGy, consistent with cumulative oxidation, microstructural reorganization, and enhanced dielectric loss processes.

**Figure 8:**
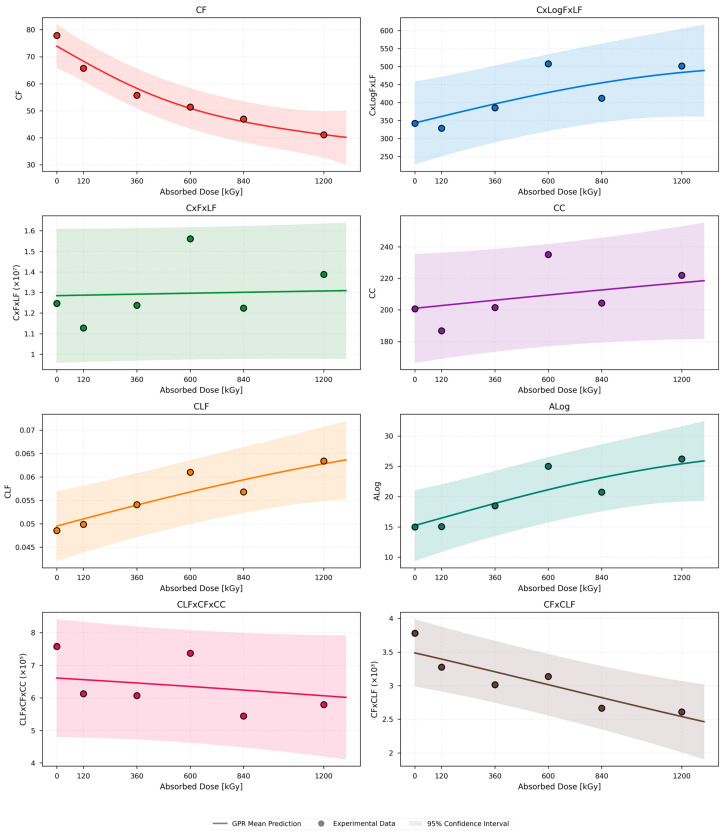
CSPE dielectric quantities dose-dependent behavior using the GPR model.

Smooth yet distinct dose-response trends across the investigated dielectric parameters are revealed by the Gaussian process regression (GPR) mean predictions, as shown in Figure 8, accompanied by broader but well-structured 95% confidence intervals. Increased variability and complexity of dielectric evolution in the CSPE jacket compared to the insulation materials are reflected by these wider intervals.

A pronounced and continuous decrease with increasing absorbed dose is exhibited by the central frequency CF, indicating a systematic shift of dielectric loss toward lower frequencies as radiation exposure progresses. This trend is consistently captured by the GPR model across the full dose range and represents the most robust and coherent aging indicator for the CSPE jacket. Moderate dose-dependent increases are displayed by dielectric loss and capacitance-related parameters, including C × LogF × LF, CC, CLF and ALog, with reduced sensitivity and larger uncertainty bands relative to EPR insulation. A partial decoupling between dielectric loss growth and underlying structural degradation in the jacket material is suggested by this behavior.

Additional complexity is exhibited by composite parameters combining multiple dielectric quantities. Weak dose-dependence and large confidence intervals are shown by the C × F × LF parameter, indicating limited utility for reliable dose estimation in CSPE. A gradual decreasing trend with dose is demonstrated by the CLF × CF × CC composite parameter, reflecting the dominant influence of the frequency shift relative to concurrent increases in dielectric loss and capacitance. The most pronounced and consistent decrease with dose among the composite metrics is exhibited by CF × CLF, further underscoring the central role of frequency-domain shifts in characterizing radiation-induced aging in CSPE jackets.

## 5. Discussion

Clear material-specific differences in the predictability of radiation aging across the investigated insulation systems are highlighted by the GPR analysis. The most reliable dose-sensitive parameters for the Black and White EPR insulation are central capacitance CC, C × F × LF, and ALog, all of which exhibit smooth monotonic growth with absorbed dose and consistently narrow confidence intervals. Strong predictive stability within the GPR framework is demonstrated by these parameters, indicating that capacitance and energy-weighted loss metrics provide the most robust representation of radiation-induced degradation in EPR materials.

The most informative indicators for the CSPE jacket emerge as frequency-related indicators, most notably CF and CF × CLF, capturing the radiation-induced shift of dielectric loss toward lower frequencies, as previously reported in the literature [31]. These frequency-dominated aging signatures are resolved without imposing restrictive functional assumptions because the GPR framework accommodates nonlinear trends and material-specific variability. This work demonstrates that AI-based modeling of derived dielectric parameters enhances material selectivity and enables clearer differentiation between EPR insulation and CSPE jacket aging under gamma irradiation, while addressing studies that emphasize the dissipation factor as a reliable aging indicator.

Highly coherent and physically consistent aging trajectories based solely on electrical measurements are yielded by applying these dielectric-derived parameters within a GPR framework to high-frequency dielectric spectroscopy data of irradiated CSPE EPR cables, with improved robustness compared to earlier single-parameter approaches applied to PVC cables for aging time and Shore D hardness evaluation [19]. The differing responses of EPR and CSPE cables to identical gamma irradiation arise from their distinct polymer chemistries and dominant aging mechanisms. Progressive increases in dielectric loss and permittivity dispersion result in EPR, which is a non-polar hydrocarbon, because oxidative chain scission creates polar carbonyl groups while reducing mechanical integrity. Low-frequency dielectric loss is enhanced in CSPE, which contains chlorine groups due to competing dehydrochlorination and cross-linking reactions that generate ionic species while mechanical integrity is partially preserved through cross-linking, leading to a decoupling of electrical and mechanical degradation pathways [8,41,42].

A consistent reduction in CF with increasing absorbed dose is identified as one of the most significant observations, indicating that radiation aging predominantly affects the low-frequency region of the dielectric response. The relative variation of dielectric loss becomes more pronounced at lower frequencies as aging progresses, and this behavior is confirmed by these results. Time-dependent modifications of electrical properties induced by gamma irradiation through molecular breakage and cross-linking have similarly been reported in investigations on ethylene propylene rubber insulation [31,35]. Enhanced dielectric dispersion associated with oxidation-related structural changes is reflected by the GPR predicted increase in CC and integrated loss-related parameters, such as ALog and C × F × LF, because these parameters inherently incorporate cumulative capacitance and loss contributions. Increased energy dissipation, higher effective conductivity, and gradual degradation of insulation performance are promoted by such changes [35]. Comparable trends have been reported for CSPE and XLPE materials, where oxidation and chain scission processes contribute to elevated dielectric losses and conduction phenomena [32].

Another important trend captured by the GPR analysis is the strong sensitivity of low-frequency, energy-weighted dielectric parameters to aging dose. Parameters combining capacitance and dielectric loss, such as C × F × LF and ALog, indicate evolving polarization mechanisms within the insulation material, including enhanced dipole mobility, charge trapping, and space charge accumulation, which collectively increase energy losses and leakage currents [9,32]. Previous studies have shown that such space charge effects, driven by oxidation and depolymerization processes, significantly influence low-frequency dielectric behavior [43,44,45,46,47]. Nevertheless, diffusion-limited oxidation phenomena in certain polymers may lead to spatially non-uniform aging, complicating the interpretation of dielectric parameter evolution and necessitating further investigation [48].

Based on the GPR-based results of this work, dielectric spectroscopy combined with machine learning analysis is recommended for field testing using material-specific indicators. For EPR insulation, capacitance- and loss-integrated parameters such as CC, ALog, and C × F × LF provide the most reliable and stable aging signatures, while for CSPE jackets, CF and CF × CLF serve as the most effective descriptors of radiation-induced dielectric evolution. The approach is particularly well suited to homogeneous polymers such as EPR subjected to dominant radiation stress in the presence of oxygen, where oxidative degradation produces distinct and predictable dielectric features. Nevertheless, important limitations must be acknowledged, including reduced diagnostic clarity for materials such as CSPE with competing degradation mechanisms, potential influence of non-uniform environmental conditions (temperature and humidity), and the need for validation under combined multi-stress aging scenarios.

## 6. Conclusions

A reliable non-destructive approach for assessing radiation-induced aging in nuclear power plant instrumentation and control cables is provided by broadband dielectric spectroscopy when combined with a GPR-based machine learning framework, as demonstrated by this study. Strong sensitivity to gamma irradiation is exhibited by the derived dielectric parameters for both ethylene propylene rubber EPR insulation and chlorosulfonated polyethylene CSPE jackets, while a clear material-specific interpretation of degradation behavior is enabled.

The GPR analysis identifies capacitance- and energy-weighted loss parameters as the most robust aging indicators for EPR insulation, reflecting a predominantly homogeneous and oxidation-driven degradation process. The aging of CSPE jackets is governed mainly by frequency-domain shifts of dielectric loss, which are effectively captured by frequency-based indicators within the machine learning framework. The inclusion of predictive uncertainty further enhances the reliability of the proposed diagnostic methodology.

The investigated dielectric variables serve as effective electrical surrogates for irradiation-induced microstructural changes traditionally evaluated by destructive mechanical testing. As such, the proposed approach supports condition monitoring, predictive maintenance, and residual life assessment without operational disruption. Future work will focus on extending the framework to the combined aging stressors and validating the methodology using in-service cable measurements.

## Figures and Tables

**Table 1:** The derived values for the EPR (Black & White) insulation and CSPE jacket, as calculated for unaged and different aging doses.

	Total Dose [kGy]	CF	C × LogF × LF	C × F × LF	CC	CLF	ALog	CLF × CF × CC	CF × CLF
BLACK Insulation	0	55,887	0.17605	670,607	0.529	0.0082	0.86	241.91	457.3
	120	53,523	0.2183	721,841	0.5908	0.0093	0.986	294.26	498.08
	360	53,477	0.3232	893,815	0.7334	0.01112	1.22856	436.076	594.61
	600	48,746	0.3831	941,160	0.8295	0.0121	1.4564	488.99	589.51
	840	46,577	0.499	1,044,607	0.9546	0.0139	1.7135	617.38	646.73
	1200	43,067	0.8116	1,332,804	1.2711	0.0172	2.3405	943.97	742.65
WHITE Insulation	0	55,938	19.07	666,515	63.8868	0.00831	1.1763	29,692	464.761
	120	52,515	21.434	717,772	62.6198	0.00953	1.33771	31,338.41	500.455
	360	51,603	25.879	852,534	65.3850	0.01102	1.62165	37,194.93	568.860
	600	48,149	29.933	935,762	68.06	0.01225	1.90035	40,152.84	589.962
	840	53,061	37.571	1,253,740	68.2741	0.01535	2.34567	55,618.78	814.639
	1200	43,485	47.635	1,405,713	73.91	0.0179	3.0631	57,733.14	781.128
JACKET Insulation	0	77,846	342.27	12,462,440	200.59	0.04854	15.0011	758,036	3778.96
	120	65,745	328.59	11,282,864	186.90	0.04986	15.0596	612,628.1	3277.85
	360	55,742	385.29	12,368,403	201.46	0.05407	18.4738	607,205.5	3014.11
	600	51,382	507.63	15,606,932	235.11	0.06103	25.007	737,238.8	3135.73
	840	46,924	412.11	12,236,330	204.26	0.05680	20.7255	544,427.2	2665.40
	1200	41,142	501.69	13,879,600	221.95	0.0634	26.215	579,336.2	2610.20

## Data Availability

The original contributions presented in this study are included in the article. Further inquiries can be directed to the corresponding author.

## Abbreviations

NPP	Nuclear power plant
IAEA	International Atomic Energy Agency
IGALL	International Generic Ageing Lessons Learned
I&C	Instrumentation and control
EPR	Ethylene propylene rubber
CSPE	chlorosulfonated polyethylene
XLPE	Crosslinked polyethylene
EVR	Extended voltage response
GPR	Gaussian process regression
EaB	Elongation at break
LIRA	Line resonance analysis
CLF	Central loss factor
CF	Central frequency
CC	Central capacitance
CxlgFxLF	Capacitance × log (frequency) × tan δ
CxFxLF	Capacitance × Frequency × tan δ
Alog	Area of capacitance x tan δ at logarithmic frequencies
